# Autochthonous infection with *Ehrlichia Canis* and *Hepatozoon Canis* in dogs from Serbia

**DOI:** 10.1002/vms3.1061

**Published:** 2022-12-29

**Authors:** Ratko Sukara, Nenad Andrić, Jelena Francuski Andrić, Darko Mihaljica, Gorana Veinović, Vladan Ranković, Snežana Tomanović

**Affiliations:** ^1^ Institute for Medical Research‐ National Institute of Republic of Serbia, University of Belgrade Group for Medical Entomology Belgrade Serbia; ^2^ Faculty of Veterinary Medicine University of Belgrade Belgrade Serbia; ^3^ Private Veterinary Clinic: ‘Vet Planet’ Belgrade Serbia

**Keywords:** dogs, Ehrlichia canis, Hepatozoon canis, polymerase chain reaction, Serbia, tick‐borne diseases

## Abstract

**Background:**

The epidemiological status concerning many canine tick‐borne diseases (TBDs) in Serbia is still insufficiently known.

**Objectives:**

Our study aimed to investigate the presence of tick‐borne pathogens of the family Anaplasmataceae and *Hepatozoon* spp., as a cause of illnesses accompanied by clinical signs that can occur in dogs with anaplasmosis, ehrlichiosis and hepatozoonosis.

**Methods:**

Dogs are included in the study based on the presence of a minimum of three clinical and/or pathological findings that could be associated with anaplasmosis, ehrlichiosis and hepatozoonosis. During the study (April–October 2018), 11 dogs met the conditions to be included in the survey. Identification of the causative agent in the blood of diseased dogs was performed by conventional PCR followed by sequencing.

**Results:**

The presence of the pathogens was confirmed in three animals (3/11, 27.3%). The presence of *Ehrlichia canis* was confirmed in 3‐month‐old female Rottweiler puppy, an 8‐year old Miniature Schnauzer female was positive for *Hepatozoon canis* infection, while 4‐year‐old mixed breed male dog was co‐infected with both mentioned pathogens. These are the first cases of autochthonous infection with *E. canis* and *H. canis* in dogs from Serbia confirmed by molecular methods.

**Conclusions:**

The results of our study indicate the importance of molecular methods to establish a reliable diagnosis of TBDs. Also, the confirmed presence of causative agents of canine monocytic ehrlichiosis and hepatozoonosis in Serbia appeals to veterinary practitioners that it is necessary to exclude the presence of those diseases in suspicious dogs.

## Graphical abstract

Dogs are included in the study based on the presence of a minimum of three clinical and/or pathological findings that could be associated with canine ehrlichiosis and hepatozoonosis. The presence of the pathogens was confirmed in three animals (3/11, 27.3%), Ehrlichia canis was confirmed in one dog, as well as Hepatozoon canis, while one dog was co‐infected with both mentioned pathogens. The confirmed presence of causative agents of canine monocytic ehrlichiosis and hepatozoonosis appeals to veterinary practitioners that is necessary to exclude the presence of those diseases in suspicious dogs

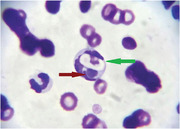

## 1 INTRODUCTION

Climate changes and expansion of the areal of vectors cause the appearance of vector‐borne diseases in dogs with a higher incidence in regions where they have appeared sporadically. Also, the occurrence of tick‐borne diseases (TBDs) caused by pathogens that were not previously confirmed in certain parts of Europe has nowadays increased (Semenza & Suk, 2018).

Previous studies have confirmed the presence of several tick‐borne pathogens in ticks collected from different localities in Serbia (Milutinović et al., 2008; Potkonjak et al., 2016; Tomanović et al., 2013a). Nevertheless, the epizootiological status concerning many canine TBDs in Serbia is still insufficiently known, while canine ehrlichiosis and hepatozoonosis have not been recognised in clinical practice to date. Three species of the family Anaplasmataceae are recognised as pathogens in dogs in Europe, namely: *Anaplasma phagocytophilum* (causative agent of canine granulocytic anaplasmosis [CGA]), *Anaplasma platys* (cyclic canine thrombocytopenia [CCT]) and *Ehrlichia canis* (canine monocytic ehrlichiosis [CME]). *A. phagocytophilum* is a confirmed zoonotic tick‐borne pathogen (TBPs), while *A. platys* and *E. canis* are potentially zoonotic (Maggi et al., 2013; Perez et al., 1996; Stuen et al., 2013). Recently, *A. platys* were confirmed as a causative agent of CCT in Serbia (Ilić Božović et al., 2021) while no clinical case of CGA and CME has been confirmed nor the DNA of the causative agent of these diseases have been detected in the blood of dogs in Serbia so far (Potkonjak et al., 2020). CME is a TBD of particular importance to the dog population in Southern Europe. The Gram‐negative bacteria *E. canis* is the main causative agent, and structures called morulae could be seen in the cytoplasm of infected leukocytes. The Brown dog tick, *Rhipicephalus sanguineus*, is considered the main vector in Europe (Mathios & Konstantina, 2017), and it is also a species that often parasitises dogs in Serbia (Potkonjak et al., 2016). CME is characterised by multiple clinical manifestations and the disease has different phases, so it is difficult to achieve a reliable diagnosis in routine clinical practice when molecular diagnostic methods are not available. Further, other TBPs (*A. phagocytophilum*, *A. platys, H. canis*, *Babesia canis*) potentially present in co‐infection, leading to an even more complex clinical presentation (Sainz et al., 2015).

Haematozoan parasite *H. canis* was recognised as a causative agent of canine hepatozoonosis in Europe. The disease is autochthonous in Southern Europe, in areas with a Mediterranean climate (Baneth, 2011). *R. sanguineus* is the main vector and the route of infection is by ingestion of an infected tick (Dantas‐Torres & Otranto, 2015). Nevertheless, transplacental transmission has also been confirmed. In predisposed dogs, the infection can lead to a life‐threatening illness although it is usually subclinical in immunocompetent animals. Fever, anaemia, lethargy, and cachexia are the most common symptoms in dogs with high parasitaemia (Baneth, 2011). There is little data on the epidemiological status of canine hepatozoonosis in Serbia. Recently, the DNA of *H. canis* is detected in the blood of one asymptomatic dog in southern Serbia (Gabrielli et al., 2015), while clinical cases of the disease in dogs have not been described in Serbia so far.

Our study aimed to investigate the presence of TBPs of the family Anaplasmataceae and *Hepatozoon* spp., as a cause of illnesses accompanied by clinical and/or pathological findings which are often present in canine anaplasmosis, ehrlichiosis, and hepatozoonosis.

## 2 MATERIALS AND METHODS

The study was conducted in 2018, during the main period of tick activity (from April to October). Sick dogs brought to the veterinary clinic for the presentation were included in the study based on the presence of a minimum of three clinical and/or pathological findings that could be associated with anaplasmosis, ehrlichiosis, and hepatozoonosis (fever, lethargy, depression, weakness, weight loss, anorexia, splenomegaly, lymphadenomegaly, petechiae and ecchymoses, epistaxis, pale mucous membranes, ophthalmological lesions, neurological disorders, lameness, anaemia, thrombocytopenia, leukopenia, lymphocytosis, hypoalbuminaemia hyperglobulinaemia, increase concentrations of alanine aminotransferase (ALT), alkaline phosphatase (ALP), or a positive rapid antibody test for *E. canis/Anaplasma* (BioNote, Korea), or point‐of‐care 4Dx Plus Test (Idexx, USA). Blood samples were taken from the cephalic vein into tubes with anticoagulant (ethylenediaminetetraacetic acid [EDTA]) for standard laboratory analysis. The samples were collected with the best practice of veterinary care and involved the informed consent of dog owners. The applied methodology does not require the consent of the ethics committee, since no specific procedure was performed on animals. Veterinarians collected all anamnestic and clinical data important for the epidemiological and clinical point of view (history of the disease, travel history, antiparasitic treatment, clinical signs, results of haematological and biochemistry analyses).

### 2.1 Molecular analysis

#### 2.1.1 DNA extraction

The 200 μl of whole blood was aliquoted and stored at 4°C before DNA extraction which was performed up to 24 h after blood sampling. The isolation of total DNA was performed using the Gene Jet Genomic DNA Purification Kit (Thermo Scientific) following the manufacturer's protocol for extraction of total DNA from mammalian blood. The extracted DNA was stored at –20°C until PCR analysis.

#### 2.1.2 Conventional PCR and sequencing

To check the presence of TBPs the multiplex PCR was performed from each sample to detect DNA of the family Anaplasmataceae and *Hepatozoon* spp., using previously published primers. Primer set: EHR16SD: GGTACCYACAGAAGAAGTCC and EHR16SR: TAGCACTCATCGTTTACAGC, were used for the amplification of 345 bp fragment of 16S rRNA which is discriminatory for species of the family Anaplasmataceae (Martin et al., 2005). The primers: HepF_for: ATACATGAGCAAAATCTCAAC and HepR_rev: CTTATTATTCCATGCTGCAG were used for amplification of the fragment of 18S small subunit rRNA gene (666 bp) for detection of *Hepatozoon spp*. (Inokuma et al., 2002). The total volume of PCR reaction was 50 μl containing: 10 μl of 5X Green GoTaq® Reaction Buffer, 1 μl of PCR Nucleotide Mix (10 mM each), 3 μl of each forward and reverse primers (10 μM concentration), 0.25 μl of GoTaq® DNA Polymerase (5 U/μl), 5 μl of tested DNA, and Molecular Biology Water up to the 50 μl. Cycling conditions were as follows: initial denaturation at 95°C for 2 min, 35 cycles at 95°C for 50 s, 55°C for 50 s, 72°C for 50 s and 72°C for 5 min. The amplification of all PCR reactions was carried out in a Veriti Thermal Cycler device (Applied Biosystems). Reaction mix without DNA as a negative control and DNA of tested pathogens as a positive control was included in parallel for all set reactions. Positive samples were subjected to singleplex PCRs with the above described conditions to prepare products for sequencing. Purification and Sanger sequencing of PCR products was performed by a Macrogen laboratory (Amsterdam, the Netherlands).

#### 2.1.3 Sequence analysis

The FinchTV software (ver. 1.4.0) was used to process obtained sequences, while the comparison of obtained with previously deposited sequences was done using the Basic Local Alignment Search Tool (BLAST) in GenBank (National Centre for Biotechnology Information, http://www.ncbi.nlm.nih.gov/BLAST). Representative sequences were deposited in the GenBank database.

## 3 RESULTS

Eleven dogs met the conditions to be included in the study (present at least three clinical and/or pathological findings associated with canine anaplasmosis, ehrlichiosis and hepatozoonoses or a positive rapid antibody test for *E. canis/Anaplasma* (BioNote, Korea), or point‐of‐care 4Dx Plus Test (Idexx, USA). Blood samples of all animals were subjected to PCR analysis, and three dogs were positive for at least one TBP (3/11, 27.3%). One female (1/11, 9.1%) was positive for the presence of DNA of the Anaplasmataceae family, while two tested males (2/11, 18.2%) were given a positive signal for the presence of *Hepatozoon* spp. DNA. One of the male dogs was positive for the presence of DNA of the Anaplasmataceae family and DNA of *Hepatozoon* spp. at the same time. Anamnestic data indicate that the positive dogs did not leave the territory of Serbia and that they live in the vicinity of Belgrade city. After analysing the obtained sequences, the presence of *E. canis* and *H. canis* was confirmed. These sequence data have been submitted to the GenBank database under accession numbers: MZ930460 (*H. canis*) and MZ931287 (*E.canis*). The analysis confirmed that obtained 16S rRNA sequences of *E. canis* were mutually identical and shared 100% sequence identity with *E. canis* isolates reported previously including dogs from the Greek island of Crete (MN922610), Philippines (KP182941, KP182942) and ticks *R. sanguineus* and *Rhipicephalus haemaphysaloides* from India (MN630202). One *H. canis* 18S rRNA sequence from the present study shares 100% sequence identity with previously reported *H. canis* isolates in dogs from Spain (AY461378), Portugal (LC018208), Israel (MH615006, MK091085, MK091087, KC138535), India (KX818220, MF797806), Pakistan (MG209591), Thailand (DQ519357), Taiwan (EU289222), West Indies (JX112783), Venezuela (DQ439543), cats from Israel (KC138531, KC138532), a red fox from Italy (KP715301), *R. sanguineus* ticks from Portugal (AB872944) and Turkey (MW684291).

### 3.1 Clinical presentations of dogs positive to *Erlichia canis* and *Hepatozoon canis*

A 3‐month‐old female Rottweiler puppy from the vicinity of Belgrade city without a travel history to the endemic area and vaccinated against infectious diseases was presented at an ordinary veterinary clinic with a high temperature (41.5°C), apathy and enlarged retropharyngeal lymph nodes. No histories of previous diseases were reported. Twenty days before, the tick was removed from the puppy by the owner, so species determination was lacking. The dog was treated with antimicrobial agents‐ceftriaxone and imidocarb dipropionate, and mineral and vitamin supplements (Mg, Fe, Cu, vitamin C, B6, B12, CoQ10, folic acid), without performing complete blood count (CBC) and other diagnostic tests. After nine days of therapy, the clinical condition worsened and the dog was referred to a hospital for small animals.

Re‐evaluated clinical examination revealed lethargy, anorexia, pale mucosa membrane, prolonged capillary refill time (3 s), and generalised lymphadenopathy without high temperature (38.4°C). CBC showed the presence of mild non‐haemolytic, normocytic, normochromic, non‐regenerative anaemia with mild neutropenia without leukocytosis and severe thrombocytopenia (Table 1). On blood and buffy coat smears granular lymphocytes were noted. Antibody point‐of‐care test (Bionote, Korea) indicated exposure to *E. canis*. Cytology of enlarged lymph nodes was not done. PCR assay followed by sequencing has confirmed the presence of *E. canis*. Antibiotic doxycycline (10 mg/kg/per os) was included in the therapy for 30 days. Clinical examination, CBC and biochemical analysis were performed once a week and after 30 days the dog recovered completely.

**TABLE 1:** Abnormal clinical, serological (point of care testing), haematological and biochemical results for the 3 PCR‐positive dogs

**Animals**	**Dog 1**, 3‐month‐old Rottweiler female	**Dog 2**, 8‐year‐old Miniature Schnauzer female	**Dog 2**, after 7 days of Prednisolone (2 mg/kg/12 h per os)	**Dog 2,** after 19 days of therapy	**Dog 2,** after 13 days of doxycycline (10 mg/kg, po, q 24 h) and immunosuppressive therapy	**Dog 3**, 4‐year‐old mixed breed male dog from shelter	**Dog 3,** after 30 days of doxycycline (5 mg/kg/po/24 h) and two doses of imidocarb dipropionate (5 mg/kg)
**Anamnesis**	History of thick bite, fever, apathy and enlarged retropharyngeal lymph nodes, treated with ceftriaxone and imidocarb dipropionate	History of idiopathic epilepsy				General weakness, anorexia	
**Clinical sings**	Lethargy, anorexia, pale mucosa membrane, prolonged capillary refill time (3 s), and generalised lymphadenopathy	General weakness and anorexia during the last six days pale mucous membranes, depression, tachycardia, tachypnea without fever	Appetite improved, weakness	Fever ( 40°C)	Normal appetite and water consumption without fever (38.9°C)	Fever (41.4°C), pale mucous membranes, tachypnea, dehydration and cachexia	
**CBC**	**First presentation**	**First presentation**	**7 days after**	**19 days after**	**32 days after**	**First presentation**	**30 days after**
RBC (6–17 × 10^12^/L)	6.1	4.29	3.56	3.51	4.17	2.9	4.4
HCT (37%–55%)	25.6	27.39	25.9	25.7	28.65	18.1	28.5
Hgb (120–180 g/L)	85	101	94	100	101	54	85
MCV (60–77 fL)	68.8	64	72.8	73.5	63	63	65
MCH (19.5–24.5 pg)	22.8	23.5	26.4	28.4	24.1	20.5	21
MCHC (20–360 g/L)	332	369	362	389	383	301	320
WBC (6–17 × 10^9^/L)	9.01	6.20	6.4	16.1	11.87	17.2	16
Ly (1.05–5.10 × 10^9^/L)	5.80	2.60	3.5	2.1	3.37	2.2	1.9
Ne (2.95–11.64 × 10^9^/L)	2.90	3.48	1.5	13.2	8.45	9.6	7.4
Eo (×10^9^/L)	/	/	/	/	/	/	/
MID (×10^9^/L; 0.0–1.8)	0.6	0.12	1.4	0.8	0.05	/	/
PLT (200–500 × 10^9^/L)	31	420	164	383	402	128	239
**Biochemical results**							
Urea (3.3–9.2 mmol/L)	/	11.97	15.70	11.55	15.69	7.5	8.0
ALP (0–190 U/L)	/	257	>1200	>1200	>1200	/	/
ALP (10.6–100.7 U/L)	/	/	/	/	/	124	41
ALT (10–50 U/L)		34.9	55	47	39	111.5	54.5
AST (10–58 U/L)		19.8	31	44	28	185.5	25.2
CK (40–254 U/L)		118	164	187	201	266	255
Albumin (28–40 g/L)		30.6	29.6	32	30.5	21.8	29.5
Globulins (21–37 g/L)						53.2	40.5
Total protein (50–80 g/L)		63.29	61	60	65	67	75
**Serology**	*E. canis*	Negative				*Dirofilaria immitis. E. canis*	
**PCR results**	*Erlichia canis*			*H. canis*	*H. canis*	*E. canis, H. canis*	*E. canis* and *H. canis*

/, not done.

*Note*: CBC were performed on haematology analyser Abacus junior Vet (Diatron, Austria) for dog 1 and dog 2, while for the dog 3 analyses were performed on Scil Vet ABC animal blood counter (Horiba, USA).

The presence of *H. canis* was confirmed in 8‐year‐old Miniature Schnauzer female, with a history of idiopathic epilepsy. The clinical signs were general weakness and anorexia during the last 6 days. Clinical examination revealed pale mucous membranes, depression, tachycardia and tachypnea without fever. CBC and blood smear analysis indicate mild, haemolytic, immune‐mediated, anaemia (IMHA), positive saline test for auto agglutination, and presence of spherocytes on the blood smear. Inclusions in leukocytes and erythrocytes were not detected on the first blood smear evaluation. No changes in leukocytes and thrombocytes were observed. Serum biochemistry analysis revealed slightly elevated urea concentration and moderate ALP activity without changes in other biochemistry parameters. Point‐of‐care 4Dx Plus Test (Idexx, USA) was negative for all selected vector‐borne diseases. For the next 7 days, prednisolone was included in the therapy, 2 mg/kg/12 h per os. The dog was clinically better, and the appetite improved, but weakness persisted without depression. Control CBC and serum biochemistry analysis showed deterioration, moderate anaemia without leukocytosis, and thrombocytopenia. Biochemistry changes included increased urea concentration without creatinine changes and elevated ALP activity (Table 1). The next 7 days prednisolone therapy continued and supplement therapy was included. During that time clinical conditions were woks and wane, and CBC was repeated every 2 days. After 19 days of therapy, fever appeared, and anaemia was more pronounced. Leukopenia was mild and platelet count was adequate (Table 1). EDTA blood samples were sent for molecular detection of vector‐borne pathogens (family Anaplasmataceae and *Hepatozoon* spp.). PCR analysis shows the positive result for *H. canis* and a negative for other tested pathogens. After 13 days on doxycycline (10 mg/kg, po, q 24 h) and immunosuppressive therapy dog was clinically better but mild non‐regenerative, haemolytic, immune‐mediated, anaemia still persisted with high urea and normal creatinine concentration. PCR analysis was repeated and the dog was for the second time positive for *H. canis*. Imidocarb dipropionate (6 mg/kg, sc,) was included in therapy and after the first dose during the next 15 days, the clinical condition was better, and the dog increased body weight by 1 kg PCR was repeated, and a weak signal was detected. Interestingly, two and a half months after the appearance of clinical signs dog was for the first time positive for *H. canis* by cytology of blood. Fifteen days later, was after the second imidocarb dipropionate treatment, the dog fully recovered.

**FIGURE 1:**
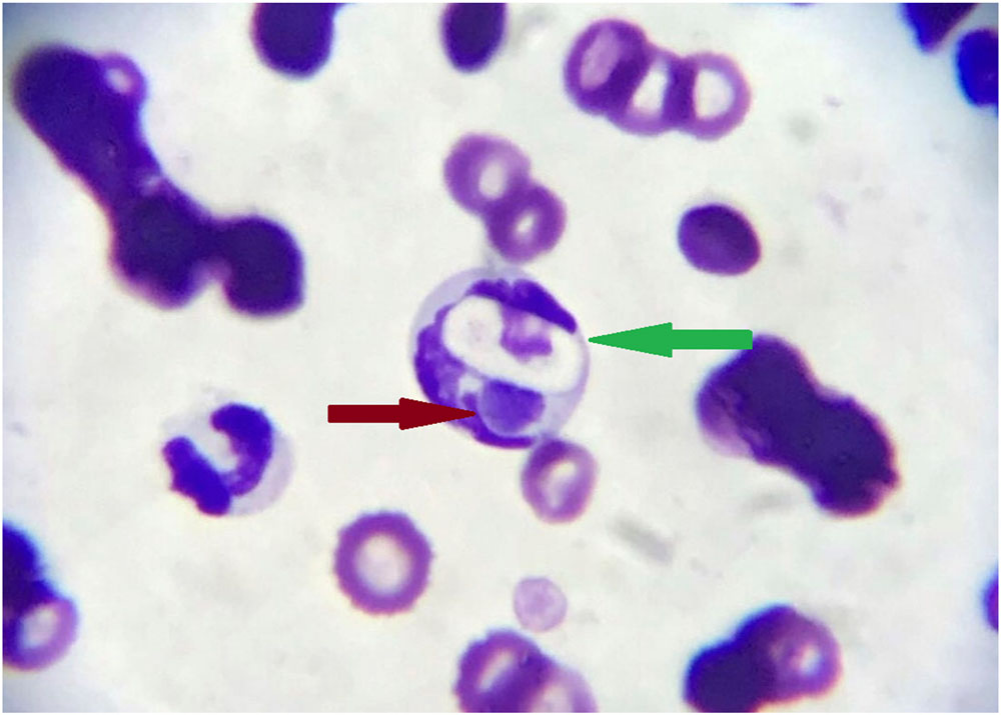
Photomicrographs of peripheral blood smear show gametocyte of *Hepatozoon canis* (green arrow) and morula of *Ehrlichia canis* (red arrow) in a mononuclear cell (Diff‐Quik × 1000)

Co‐infection with *E. canis* and *H. canis* was confirmed in 4‐year‐old mixed breed male dog from the shelter with general weakness and anorexia. Clinical examination revealed fever (41.4°C), pale mucous membranes, tachypnea, dehydration and cachexia. CBC indicates severe anaemia and thrombocytopenia with mild leukocytosis and eosinophilia. Serum biochemistry analysis revealed an elevated concentration of ALT, ALP, aspartate aminotransferase (AST) and creatine kinase (CK). Albumin concentration was while globulins were elevated (Table 1). Idexx 4D point‐of‐care test was positive for the presence of antibodies against *E. canis*, and antigen of the *Dirofilaria immitis*. Blood smear evaluation reveals the presence of ellipsoidal inclusion in mononuclear cells which looks like on *H. canis* gametocytes as well as the presence of morulae (Figure 1). PCR analysis was required to identify inclusions in mononuclear cells. Co‐infection with *E. canis* and *H. canis* was confirmed. Antimicrobial treatment with imidocarb dipropionate (5 mg/kg, twice, 15 days apart) and doxycycline (5 mg/kg/bid for 30 days) was conducted. After treatment, inclusions were not detected on the blood smear. Control CBC indicated the presence of mild anaemia, without changes in platelets. The results of PCR remained positive for *E. canis* and *H. canis*. The dog was clinically better but the therapy was continued for another month. After that period dog was the third time, tested by PCR, and the results were again positive for *E. canis* and *H. canis*, although clinical or pathological findings of ehrlichiosis and hepatozoonosis were no longer present.

## 4 DISCUSSION

In the present study, infection with *E. canis* and *H. canis* in dogs from Serbia was confirmed for the first time by molecular methods. Also, one co‐infection with *E. canis* and *H. canis* was recorded. Positive animals have not left the territory of Serbia, which indicates an autochthonous infection.

*Ehrlichia*, *Anaplasma*, *Neorickettsia* and *Wolbachia* are genera classified in the family Anaplasmataceae (Dumler et al., 2001), while three species within these genera are identified as significant dog pathogens in Europe: *E. canis, A. phagocytophilum, A. platys*. *E. canis* is a TBP recognised as a causative agent of CME which is one of the major canine TBDs in southern Europe and has an endemic character in Mediterranean countries. In Europe, brown dog tick *R. sanguineus* is considered as the main vector of *E. canis* (Sainz et al., 2015). Previous research has confirmed that *R. sanguineus* most often infests dogs in our country as well (Bogunović et al., 2018; Potkonjak et al., 2016). Even though previous seroepidemiological studies conducted in Serbia have confirmed the exposure of dogs to *E. canis* (Bogićević et al., 2017; Potkonjak et al., 2013), clinical cases of CME or molecular confirmation of the causative agent have not been reported before (Kovačević Filipović et al., 2018). Also, the presence of *E. canis* in *R. sanguineus* ticks collected from dogs in Serbia has not been confirmed so far (Potkonjak et al., 2016).

CME is characterised by three stages: acute, chronic and subclinical. Thrombocytopenia occurs in over 80% of all cases (Mathios & Konstantina, 2017), and our results fit this pattern. Both positive dogs in our study had thrombocytopenia. Although thrombocytopenia is present in most animals with CME, it occurs in many other vector‐borne diseases and other haemostasis disorders indicated their important role not just in coagulation but also in inflammation. Severe pancytopenia as a consequence of bone marrow hypoplasia which is present in the chronic phase of CME can be fatal (Sainz et al., 2015). Different phases and multiple clinical manifestations of CME, as well as potential co‐infections with other TBPs (*Babesia spp., H. canis, A. phagocytophilum, A. platys*) complicate reliable diagnosis. Even though routine haematological, serological and cytological diagnostic techniques are of great importance for CME, molecular methods (PCR, qPCR) are often necessary for a definitive diagnosis. Finding morulae of *E. canis* in monocytes during the microscopic examination of Romanowsky‐type stained blood smears is not reliable enough to diagnose CME, because the method has low sensitivity and specificity concerning the possible presence of artefacts or other inclusions and more often could be seen in acute phase of infection. *E. canis* morulae were not undoubtedly identified in the stained blood smear of the diseased dog in our study in which mono‐infection was confirmed by PCR, while they were clearly visible in the blood smears of the dog in co‐infection with *E. canis* and *H. canis* (Figure 1). Although serological methods (indirect fluorescent antibody‐IFA and enzyme‐linked immunosorbent assays‐ ELISA) are commonly used in routine practice to diagnose CME, these methods indicate exposure to *E. canis* rather than active infection. Both dogs in our study were serologically positive for the presence of *E. canis*. The ambiguity of serological assays maybe also the appearance of cross‐reactivity among *E. canis* and other species of the Anaplasmacataceae family (*Ehrlichia chaffeensis, Ehrlichia ewingii, A. phagocytophilum*) (Qurollo et al., 2021). Only PCR assay with high specificity and sensitivity is essential to detect DNA of *E. canis* indicating active infection. Also, a significant advantage of PCR is the possible detection of DNA of *E. canis* before seroconversion (René‐Martellet et al., 2015), which is important in clinical practice to apply timely therapy and prevent severe cases of the disease.

Autochthonous canine hepatozoonoses have been reported in Southern Europe, mainly in the countries with a Mediterranean climate where *R. sanguineus* is widely distributed (Baneth, 2011; Pacifico et al., 2020). A recent study confirmed autochthonous infection with *H. canis* in dogs from Germany indicating that canine hepatozoonoses could be expected also in the countries out of the Mediterranean basin (Helm et al., 2020). Based on the epidemiological situation in the region, it is quite certain that the importance of canine hepatozoonoses in our country was underestimated. The results of a recent study on the red foxes in Serbia support this statement. A relatively high number of positive animals for the presence of *H. canis* was confirmed. The overall prevalence of 61.2% was recorded by molecular methods (Juwaid et al., 2019). Authors suggested possible spillover from the sylvatic cycle to domestic dogs by competent vectors since foxes are recognised as hosts for *R. sanguineus* ticks in Serbia (Tomanović et al., 2013b), a species that is at the same time most abundant on dogs in Serbia (Bogunović et al., 2018; Potkonjak et al., 2016).

Further studies are needed to elucidate the paths of transmission concerning *H. canis* in Serbia. So far, the DNA of *H. canis* is proven in the blood of one asymptomatic dog in southern Serbia (Gabrielli et al., 2015) while the parasite has not been confirmed in the competent tick vector so far (Potkonjak et al., 2016). In the present study, the clinical case of canine hepatozoonosis has been confirmed by conventional PCR followed by sequencing for the first time. It was not possible to notice *H. canis* gametocytes in the blood of the diseased dog at the onset of illness, probably due to a small number of infected blood cells and limited sensitivity of microscopic examination. In this particular case, the importance of molecular diagnostics in the early detection of *H. canis* infection is emphasised. Adequate therapy after the confirmed presence of *H. canis* by PCR gave results and led to the complete recovery of the diseased dog. Co‐infection with two or more pathogens commonly complicates the clinical presentation of the diseases and special treatment protocol is needed in co‐infected animals. A great advantage of PCR compared to other diagnostic methods is the ability to detect several pathogens simultaneously. In the present study, one dog was infected with *E. canis* and *H. canis* at the same time. This is not an unexpected finding since mentioned pathogens share the same competent tick vector and occasionally infect even the same blood cells (Baneth et al., 2015).

## 5 CONCLUSION

In the present study, for the first time in Serbia, autochthonous CME and canine hepatozoonoses have been confirmed. Molecular assays have unequivocally confirmed the presence of DNA of *E. canis* and *H. canis* in dogs with clinical and pathological features associated with those TBDs. One dog was co‐infected with both mentioned pathogens. Based on the obtained results, it is reasonable to expect the endemic occurrence of CME and canine hepatozoonosis in Serbia. We appeal to veterinary practitioners to include in the differential diagnosis these TBDs in suspicious dogs. Co‐infection with different TBPs should also be considered.

## AUTHOR CONTRIBUTIONS

Ratko Sukara: writing – original draft preparation (lead); investigation (lead); formal analysis (equal). Nenad Andrić: conceptualisation (equal); investigation (equal); writing – original draft preparation (equal); funding acquisition (equal). Jelena Francuski Andrić: investigation (equal); writing – original draft preparation (equal). Darko Mihaljica: formal analysis (equal); writing – review and editing (equal). Gorana Veinović: formal analysis (equal); writing – review and editing (equal). Vladan Ranković: investigation (equal). Snežana Tomanović: conceptualisation (equal), funding acquisition (lead), formal analysis (equal), supervision (lead); writing – original draft preparation (equal).

## CONFLICT OF INTEREST

The authors have no conflict of interest to declare.

### ETHICS STATEMENT

The authors confirm that the ethical policies of the journal, as noted on the journal's author guidelines page, have been adhered to. No ethical approval was required as this study required no specific procedure on animals or animal experiments.

### PEER REVIEW

The peer review history for this article is available at https://publons.com/publon/10.1002/vms3.1061

## Data Availability

The data that support the findings of this study are available from the corresponding author upon reasonable request.
